# Can the Molar Insulin: C-Peptide Ratio Be Used to Predict Hyperinsulinaemia?

**DOI:** 10.3390/biomedicines8050108

**Published:** 2020-05-03

**Authors:** Lynda Guildford, Catherine Crofts, Jun Lu

**Affiliations:** 1 School of Public Health and Interdisciplinary Studies, Faculty of Health and Environmental Sciences, Auckland University of Technology, Auckland 0627, New Zealand; lynda.guildford@aut.ac.nz; 2 School of Science, Faculty of Health and Environmental Sciences, Auckland University of Technology, Auckland 1010, New Zealand; 3 Human Potential Centre, Faculty of Health and Environmental Sciences, Auckland University of Technology, Auckland 0632, New Zealand; 4 Maurice Wilkins Centre for Molecular Biodiscovery, Auckland 1010, New Zealand; 5 Institute of Biomedical Technology, Auckland University of Technology, Private Bag 92006, Auckland 1142, New Zealand; 6 College of Life and Marine Sciences, Shenzhen University, Shenzhen 518071, China; 7 College of Food Engineering and Nutrition Sciences, Shaanxi Normal University, Xi’an 710119, China

**Keywords:** hyperinsulinaemia, insulin, c-peptide, oral glucose tolerance test, correlation, prediction

## Abstract

Hyperinsulinaemia is the precursor to numerous metabolic disorders. Early diagnosis and intervention could improve population health. Diagnosing hyperinsulinaemia is problematic because insulin has a very short half-life (2–5 min). It is theorised that c-peptide levels (half-life 20–30 min) would be a better proxy for insulin due to both hormones being released in equimolar amounts. However, the correlation between c-peptide and insulin levels is unknown. We aim to identify their correlation following a four-hour oral glucose tolerance test (OGTT). Data were obtained from records of routine medical care at St Joseph’s Hospital, Chicago, IL, USA, during 1977. Two hundred and fifty-five male and female participants aged over 20 years undertook a four-hour OGTT with plasma glucose, insulin and c-peptide levels recorded. Correlation was assessed with Pearson’s correlation. There was a weak correlation between insulin and c-peptide, which increased to moderate across the four-hour OGTT (r = 0.482–0.680). There was no significant change in this relationship when data was subdivided according to either the WHO glucose status or Kraft insulin response. Although there was a correlation between insulin and c-peptide, it was too weak to recommend the use of c-peptide as an alternative biomarker for the diagnosis of hyperinsulinaemia.

## 1. Introduction

Hyperinsulinaemia is becoming recognised as the primary driver of a plethora of metabolic disorders, especially insulin resistance and type 2 diabetes, but also certain cancers and dementias and cardiovascular disease [1,2,3,4]. Diagnosing hyperinsulinaemia could assist in the early diagnosis and management of many metabolic disorders.

Measuring insulin is challenging due to the pulsatile nature of its secretion [5], and because up to 80% of insulin is metabolised during the first pass metabolism in the liver [6]. Pro-insulin is released from the pancreas and is then cleaved to produce insulin and c-peptide in equimolar amounts [7,8,9]. The pharmacokinetics of c-peptide and insulin in peripheral blood are different, with insulin being metabolised in the liver and c-peptide in the kidneys [10,11]. This difference in metabolism between c-peptide at 20–30 min should be the same molar amount as that of insulin at 2–5 min, making c-peptide a potentially more dependable marker for both the quantification of insulin in peripheral blood plasma levels and beta-cell function [7,10]. Cerutti et al. suggested that the use of c-peptide as a proxy measurement of insulin on account of its molar relationship may not be valid due to the difference in their kinetics and half-life [1]. However, the combination of insulin’s instability and the c-peptide’s longer half-life has led to significant interest in using c-peptide as a proxy for insulin in measures of insulin resistance [3,12,13,14]. C-peptide is used as a biological marker in its own right in the measurement of excessive insulin both clinically and forensically [13,15]. As diabetes is known to be a high-risk factor for developing cancers, c-peptide has been used as a diagnostic marker for insulin in the identification of risk factors for cancer [16,17,18].

However, while there is a plethora of information on the use of c-peptide as a proxy for insulin, there remains a paucity of data on the direct correlation between insulin and c-peptide levels. This research may have been hampered by the lack of a multi-sampled c-peptide analyses. Between the 1970s and 1990s, seminal work was undertaken on oral glucose tolerance testing (OGTT) with insulin assays on more than 15,000 individuals [2,19]. A unique subset, predominately from 1977, also underwent concurrent assessment of c-peptide levels. Although several articles discussed the relationship between c-peptide and insulin, only one specifically discussed the correlation between the two hormones [5]. Given the paucity of literature relating to this specific topic, it appears that this is a novel investigation. Hence, levels of insulin against c-peptide at the various time points of a four-hour OGTT were analysed for their correlation in order to find out whether c-peptide levels can be used to predict insulin responses and the early diagnosis of hyperinsulinaemia.

## 2. Materials and Methods

### 2.1. Participants

Between the 1970s and 1990s, as part of routine medical practice, patients and healthy volunteers, referred by their medical practitioners, undertook a multi-sampled OGTT with insulin assays at St Joseph’s Hospital, Chicago, IL, USA [2]. A subset of individuals (*n* = 422) had concurrent quantification of c-peptide levels. This latter data set was assessed to explore the relationship between c-peptide and insulin responses. The data collected from the subset included the following: plasma glucose, insulin and c-peptide levels, as well as age, gender, height, weight and body mass index (BMI) (Table 1).

### 2.2. Inclusion Criteria

From this initial data subset, only participants over the age of 20 years were included, which resulted in a total of 335 participants (female = 217, male = 118).

### 2.3. Exclusion Criteria

Exclusion criteria removed participants with probable but significant confounding conditions, including a BMI of less than 18.0 kg/m^2^, participants who identified as pregnant or post-partum, and those with missing information. A further 63 participants were excluded due to possible confounding pathological conditions. These were participants with insulin levels ≥ 250 µU/mL and/or c-peptide levels ≥ 30 ng/mL. Participants showing results of high c-peptide levels but low insulin levels could indicate kidney disease as c-peptide is excreted via the kidneys [9]. Conversely, participants showing results of high levels of insulin and lower levels of c-peptide suggests hepatic dysfunction or could indicate exogenous insulin, as insulin is metabolised via this organ [10]. The final number of participants was 255; females = 164, males = 91.

### 2.4. Study Protocol

The study protocol was the same as that outlined by Crofts et al. [2]. Participants were required to fast overnight (10–16 h). Venous blood was then taken to obtain baseline glucose, insulin and c-peptide levels. Following this procedure, participants were required to ingest 100 g of glucose in water. Further venous blood samples were taken at 30 min and 60 min and then hourly for three to five hours as directed by the physician. Blood samples were then analysed using the glucose oxidase method, [2]. Insulin levels were determined using the Phadebus insulin radioimmunoassay test (RIA) [2]. C-peptide levels were analysed using a radioimmunoassay test, though it should be noted that sensitivity variations occurred at different concentrations [20].

### 2.5. Ethics

Analysis of the data subset was granted ethical approval by Auckland University of Technology Ethics Committee (AUTEC) on 14 November 2018. Approval reference: 18/408.

### 2.6. Participant Classification

Glucose tolerance was defined by the World Health Organization (WHO) criteria: normal glucose tolerance (NGT), impaired fasting glucose (IFG), impaired glucose tolerance (IGT), and diabetes mellitus (DM) [21]. Insulin tolerance was defined by the Kraft insulin patterns I–V as according to Crofts [2]. Table 1 summarises the characteristics of the participants; reflecting age, BMI, WHO glucose status and Kraft insulin response pattern. The *p*-values for age and BMI for gender comparison were not statistically significant (0.524 and 0.111), and the Cohen’s d value showed a weak effect (0.085 and 0.21, respectively, are not shown here).

### 2.7. Calculations and Statistical Analysis

Continuous variables were summarized as mean ± standard deviation. Independent t test tested for significance in mean values between groups. The results were considered statistically significant at a *p*-value less than 0.05. The area under the curve (AUC) was calculated by the trapezoidal rule. Scatterplots were undertaken for c-peptide and insulin for each of the following time points; 0, 30, 60, 120, 180, and 240 min to assess the correlation between variables. A sub-analysis of scatterplots was also undertaken after the groups were divided according to the WHO glucose status or Kraft response patterns. For all scatterplots, the paired Pearson’s correlation was undertaken. All statistical analysis was undertaken either using Microsoft Excel 2016 or IBM SPSS Statistics 25.

## 3. Results

**Figure 1:**
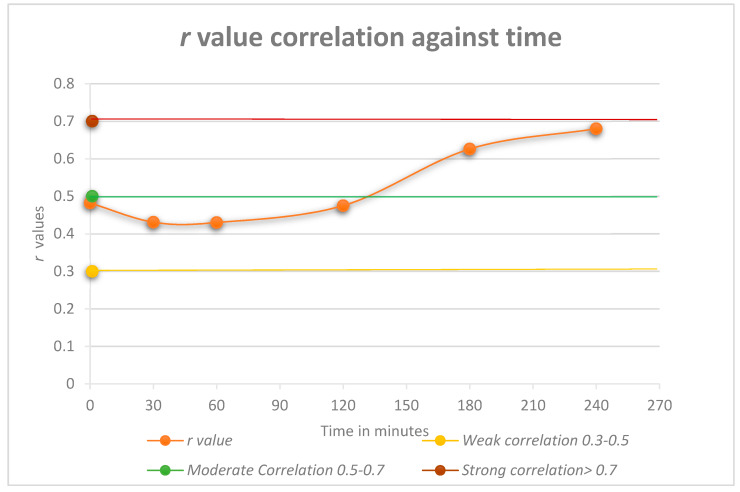
Pearson correlation *r* values against time. Correlation strength can be interpreted as: Weak = 0.3–0.5, Moderate = 0.5–0.7, Strong = > 0.7.

This study investigated the correlation between c-peptide and insulin during a four-hour OGTT. As shown in Figure 1, at the first time point (0 min), there was an initial weak correlation of r = 0.482. The correlation then weakened (r = 0.431−0.430) but strengthened at the 120 min time point to r = 0.475. The latter time points showed an increase in the correlation to a moderate value (r = 0.626 and 0.680, respectively).

The data were further sub-divided according to the Kraft insulin response pattern and separately by the WHO glucose status to identify if either glucose or insulin impacted on the correlation of c-peptide against insulin concentrations on the results. There was some difference in the range of the *r* value for the WHO glucose status, but, overall, these were similar to the original correlation values for c-peptide against insulin concentration. The *r* value for normal glucose tolerance (NGT) peaked at 180 min (*r* = 0.505), and diabetes mellitus (DM) had a higher starting *r* value (*r* = 0.560), which then dropped sharply at 30 min (*r* = 0.289) only to continue to steadily increase at 180 min (NGT *r* = 0.168–0.376, IGT *r* = 0.337–0.773, DM *r* = 0.560–0.663).

**Figure 2:**
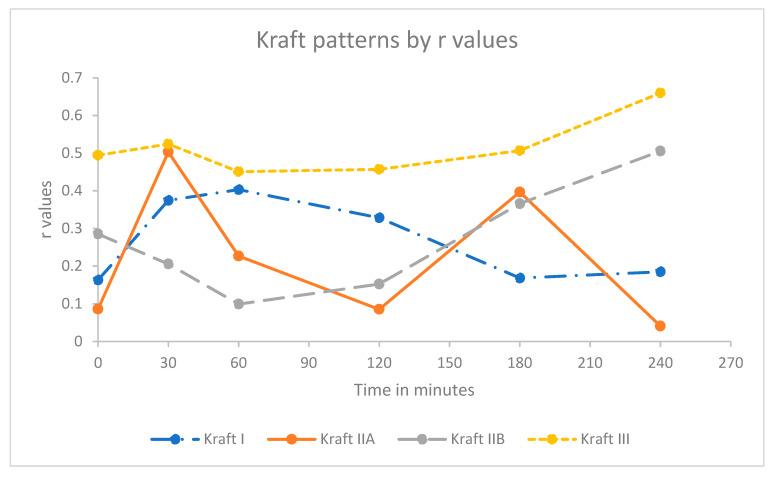
Kraft insulin response pattern r values against time. Due to the limited number of participants, Kraft pattern IV and V were not used in further analysis.

The trend of a weak correlation at the early time point for the Kraft insulin response pattern was similar to that observed with the c-peptide against insulin concentrations with two notable exceptions. Kraft pattern I demonstrated a weak increasing correlation up to 60 min (*r* = 0.403) trending downwards thereafter to 240 min (*r* = 0.185). By contrast, Kraft pattern IIA demonstrated two defined peaks at 30 min and 180 min (*r* = 0.503 and 0.366, respectively) separated by a significant trough, with the lowest point being at 120 min (*r* = 0.085), as seen in Figure 2.

Kraft pattern IIA contained two defined subgroups based on the summed levels of insulin at two and three hours. These are defined as follows: Fasting insulin ≤ 50 µU/mL, 30 min or 1 h peak with 2 + 3 h sum ≥60, <100 µU/mL or fasting insulin 31–50 µU/mL, 30 min or 1 h peak with 2 + 3 h sum <60 µU/mL. Further analysis of these two subgroups was considered to assess the impact on results; however, upon closer inspection, it was identified that all participants were of the same subgroup. Kraft pattern IV and V had insufficient data to be further considered within any further sub analysis.

**Figure 3:**
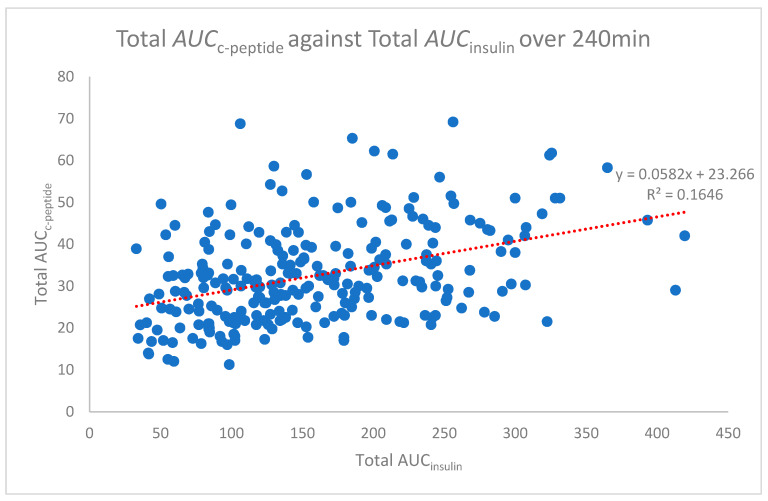
Total area under the curve c-peptide against insulin over 240 min.

**Figure 4:**
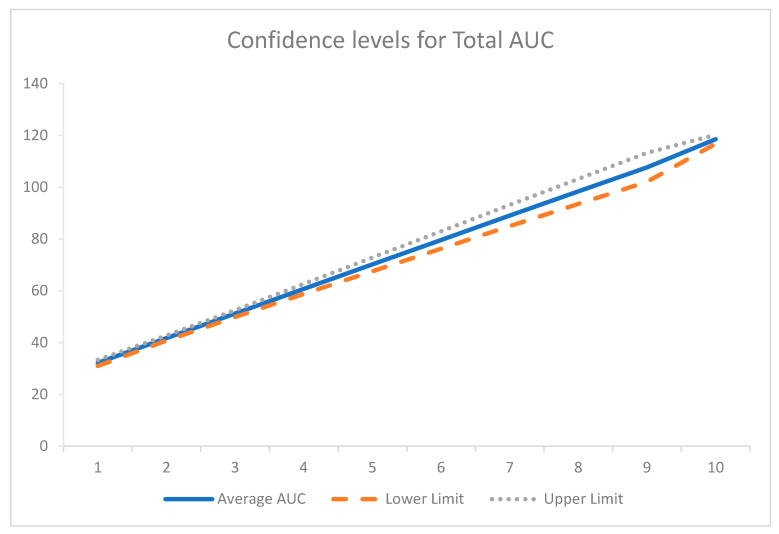
Confidence intervals for total AUC.

The area under the curve analysis was determined using the trapezoidal rule for each time point for all participants. Pearson’s correlation relating to this data was undertaken to assess the relationship of the area under the curves for insulin and c-peptide. The total area under the curve (Total AUC_c-peptide_ against AUC_insulin_) analysis was also conducted. The average total AUC for 240 min of the OGTT is represented in Figure 3. Pearson’s correlation for the total AUC analysis demonstrated only a weak correlation (*r* = 0.406), though, when 95% confidence intervals were applied to Figure 3, there was little deviation from the average (Figure 4).

Using the methods of Genazzani et al. and Lunger et al. [22,23], the results were also stratified by fasting insulin levels (<7 µU/mL, 7–12 µU/mL and >12 µU/mL) to identify if this affected the Pearson correlation *r* value and overall results. There was negligible impact demonstrated, as the lower levels had no correlation or a slightly negative correlation (*r* = 0.0019 and −0.0088, respectively) with the >12 µU/mL having an r value of *r* = 0.4531.

**Figure 5:**
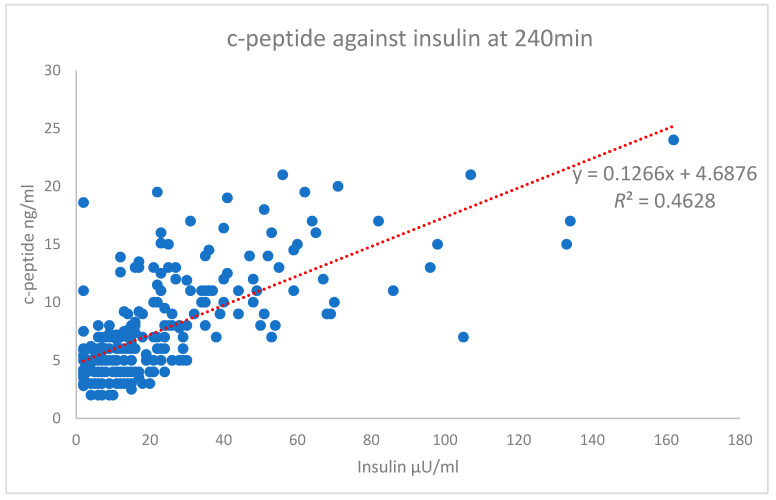
Insulin *vs*. c-peptide at 240 min.

The scatterplot of the independent variables’ c-peptide against insulin at the 240 min time point is shown in Figure 5. The Pearson’s correlation of this time point is on the high end of the moderate correlation with an r value of 0.680 (strong > 0.7). The test results highlight the difference between the Pearson’s correlation of the total AUC r value (0.406), which shows a weak correlation (Figure 3). In comparison, the overall data for each time point (Figure 1) starts with a high-end weak r value (0.482) up to a high-end moderate value (0.680). Only the 240 min time point is shown in Figure 5.

## 4. Discussion

This study investigated the correlation between blood c-peptide and insulin levels to identify whether c-peptide could be used to diagnose hyperinsulinaemia. In theory, c-peptide is a superior biomarker compared to insulin as it has a longer half-life [5,10].

The combined results for the dataset demonstrated that there was a weak to moderate correlation between blood c-peptide and insulin levels. Although weakest across the first two hours of the four-hour OGTT (r ≤ 0.500), the correlation did increase during the last two hours to a moderate correlation (0.500 ≤ r ≤ 0.700). These results were not anticipated, as previous studies demonstrated that these two molecules are released in equimolar amounts [10,11]. As previously stated, there is little research relating to the correlation of insulin and c-peptide. Anoop et al. discussed that they had found a strong correlation between insulin and c-peptide, but no values were given [5]. The majority of the work recommending c-peptide as an alternative biomarker for insulin relates to the c-peptide to glucose ratio, indicating beta-cell function rather than hyperinsulinaemia [24].

The data was then subdivided by the WHO glucose status and Kraft pattern to identify if this would impact on the correlation between insulin and c-peptide. As noted in the results section, the Kraft pattern results were similar to those of the WHO diabetes classification with regards to the r value, though only four of the six patterns were plotted due to insufficient data for Kraft patterns IV and V.

Previous studies have used upper and lower limits of fasting insulin to identify those with insulin resistance [22,23]. Using this analysis found little to no difference in the r value compared to the previous Pearson correlation analysis undertaken of c-peptide against insulin. This may be because the previous analysis was looking at insulin resistance and not the correlation between c-peptide and insulin.

The lack of correlation in the results may be attributable to the proportions of each sample within the groupings for WHO diabetes classification and Kraft pattern compared to the overall dataset described by Crofts et al. [2]. They noted that 24% of those in Kraft I also classified as NGT, while this subset had 48% [2]. Other results were within ±10% of Crofts et al. results. The exception was for IGT and Kraft IIB and Kraft III; these were 22% and 67%, respectively, for Crofts et al., but 35% and 35%, respectively, for this data set. This variation could be explained by the difference in the size of the data set under investigation as it comprises only 3.1% of the Crofts et al. data set. This difference may also have been responsible for the unusual r value result in Kraft IIA but would require further investigation.

### 4.1. Limitations

There are several limitations to this research, primarily related to the participants. These included limited information relating to the reason for test referral, ethnicity, socioeconomic status, and co-morbidities; especially those surrounding hepatic and renal disease, which could impact insulin and/or c-peptide clearance. Another limitation is the analytical method of c-peptide. Though it was tested using RIA, there is no information relating to the concentration of the c-peptide prior to analysis or analysis sensitivity, except that “variations occur at different concentrations” [20] (p. 218). The testing of c-peptide levels at this time had issues. Human c-peptide is a relatively small molecule with 31 amino-acids [25,26]. Early assays used 131I Tyr-c-peptide as a marker in the radioimmunoassay. This may be porcine or bovine in origin. It was identified that these substances differed from human c-peptide and did not give good results. Improvements in the methodology and availability of biosynthetic human c-peptide by the mid to late 1980s helped to improve inconsistencies in results [26]. Unusually low insulin levels within the data at various intervals may have been due to the haemolysis of the blood samples, either when initially taken or during storage, which caused irregularities within the data set. Overall, this suggests that there is inadequate generalisability of the results.

Bias may be present if one or more ethnic groups had a predisposition to developing specific metabolic diseases, or a socioeconomic group that has a greater chance of developing specific metabolic diseases related to hyperinsulinaemia. Groups that may be more predisposed to hyperinsulinaemia are those who may have West African origins [14], Asian origins, both Indian and Oriental, as well as Hispanic Americans and indigenous populations such as the Pima Indians and other native Indian populations [5,25,27]. Consideration should also be given to Aboriginal peoples in the Pacific region, including Māori in New Zealand [27]. In the United States Chicago area in the 1980s, the population had a large Black (39.8%) and Hispanic/Latino (14%) population [28].

These results are important because this appears to be the first time that the high frequency of sampling of c-peptide was undertaken. It has a good sample data set with c-peptide results available to be compared with that of insulin taken at the same time. However, even now there are a large variety of tests available for the testing of both c-peptide and insulin, with each giving differing results for the same test, leading to a call for the standardisation of these tests [29,30]. This would then enable standardised tables for diagnosis purposes.

### 4.2. Future Research

Future research could look towards the use of bioequivalence rather than a direct comparison of blood levels. Looking into the shape of a graph that plots insulin and c-peptide release against that of time may offer an alternative method to diagnose hyperinsulinaemia. This research should be repeated using modern analytics techniques to determine if this would improve the correlation between c-peptide and insulin. The use of c-peptide: glucose ratio should also be investigated as a marker for hyperinsulinaemia. Whichever type of testing is implemented, a standardisation for testing both insulin and c-peptide needs to occur globally to enable the development of a universal method of testing.

## 5. Conclusions

There is significant potential for c-peptide to be used in the early diagnosis of hyperinsulinaemia and thereby type 2 diabetes, because it is a more stable molecule. While this data set has identified that there is a correlation between insulin and c-peptide, it also demonstrates that this is not a strong enough correlation at this time to enable c-peptide to be used exclusively in place of insulin. Furthermore, as there is currently no standardised method of analysis for either insulin or c-peptide, and results are dependent on the brand of analysis used, when diagnosing hyperinsulinaemia, insulin should be considered the agent of choice until the relationship between c-peptide and insulin is better elucidated.

## Figures and Tables

**Table 1:** Table of participant characteristics.

		Total	Men	Women	*p*
*n*		255	91	164	
Age (years)		46.2 (16.7)	47.1 (15.4)	45.7 (17.4)	0.52
BMI (kg/m^2^)		25.2 (5.16)	25.9 (3.80)	24.9 (5.75)	0.11
WHO glucose status					
	Diabetes mellitus	103 (40%)	46 (50.5%)	57 (34.8%)	
	Impaired glucose tolerance	68 (26.7%)	25 (27.5%)	43 (26.2%)	
	Impaired fasting glucose	1 (0.4%)	0	1 (0.6%)	
	Normal glucose tolerance	83 (32.5%)	20 (22%)	63 (38.4%)	
Kraft Pattern					
	Kraft I (Normal insulin)	59 (23.1%)	18 (19.8%)	41 (25%)	
	Kraft IIA (Borderline)	32 (12.5%)	11 (12.1%)	21 (12.8%)	
	Kraft IIB (Hyperinsulinaemia)	48 (18.8%)	15 (16.5%)	33 (20.1%)	
	Kraft III (Hyperinsulinaemia)	100 (39.2%)	39 (42.9%)	61 (37.2%)	
	Kraft IV* (Hyperinsulinaemia)	2 (0.8%)	1 (1.1%)	1 (0.6%)	
	Kraft V* (Hypoinsulinaemia)	14 (5.5%)	7 (7.7%)	7 (4.3%)	

Frequency data are reported as n (%), otherwise mean (SD). Effects sizes for Cohen’s d are interpreted as: large > 0.5, moderate 0.3–0.5, and small ≤ 0.2. * Due to the limited number of participants, Kraft pattern IV and V were not used in further analysis.
